# MonoAMP: Adaptive Multi-Order Perceptual Aggregation for Monocular 3D Vehicle Detection

**DOI:** 10.3390/s25030787

**Published:** 2025-01-28

**Authors:** Xiaoxi Hu, Tao Chen, Wentao Zhang, Guangyi Ji, Hongxia Jia

**Affiliations:** School of Mathematics and Computer Science, Shaanxi University of Technology, Hanzhong 723001, China; huxiaoxi@snut.edu.cn (X.H.); zhangwentao@snut.edu.cn (W.Z.); jiguangyi@snut.edu.cn (G.J.); jiahongxia@snut.edu.cn (H.J.)

**Keywords:** autonomous driving, monocular 3D object detection, cross-dimensional attention, multi-scale fusion, multi-order aggregation

## Abstract

Monocular 3D object detection is rapidly emerging as a key research direction in autonomous driving, owing to its resource efficiency and ease of implementation. However, existing methods face certain limitations in cross-dimensional feature attention mechanisms and multi-order contextual information modeling, which constrain their detection performance in complex scenes. Thus, we propose MonoAMP, an adaptive multi-order perceptual aggregation algorithm for monocular 3D object detection. We first introduce triplet attention to enhance the interaction of cross-dimensional feature attention. Second, we design an adaptive multi-order perceptual aggregation module. It dynamically captures multi-order contextual information and employs an adaptive aggregation strategy to enhance target perception. Finally, we propose an uncertainty-guided adaptive depth ensemble strategy, which models the uncertainty distribution in depth estimation and effectively fuses multiple depth predictions. Experiments demonstrate that MonoAMP significantly enhances performance on the KITTI dataset at the moderate difficulty level, achieving 16.80% ${AP}_{3D}$ and 24.47% ${AP}_{BEV}$. Additionally, the ablation study shows a 3.78% improvement in object detection accuracy over the baseline method. Compared to other advanced methods, MonoAMP demonstrates superior detection capabilities, especially in complex scenarios.

## 1. Introduction

Three-dimensional object detection plays an essential role in environmental understanding and trajectory forecasting for autonomous vehicles. Presently, the top-performing 3D detectors primarily utilize 3D LiDAR sensors thanks to their capability to deliver highly accurate depth measurements of the surroundings. However, LiDAR-based systems are prohibitively expensive, hindering their widespread adoption. In contrast, monocular cameras have garnered increasing attention across various application scenarios due to their cost-effectiveness and simplicity in deployment.

Monocular 3D object detection faces several significant challenges. Although existing methods have made optimizations in feature extraction, feature fusion, and bounding box regression, several critical issues persist: (1) Limited receptive field: Monocular 3D object detection significantly depends on feature extractors to capture spatial information. However, many existing methods [1,2,3,4] are constrained by the limited receptive field of their backbone networks. A small receptive field prevents the model from capturing long-range dependencies and contextual information, particularly for distant or occluded objects. In scenarios where objects are far apart or partially hidden (e.g., in urban driving scenes), these methods struggle to capture global features, leading to inaccuracies in object localization and depth estimation. (2) Insufficient multi-scale feature capture: Driving scenes inherently involve objects at varying sizes and distances, making multi-scale feature extraction essential. However, existing methods [5,6,7,8] often fail to effectively capture features at different scales due to inadequate handling of spatial correlations across resolutions. These methods typically rely on single-scale feature extractors or shallow architectures, which fail to capture the full range of object sizes. This results in poor performance in complex scenes, particularly when handling objects with significant scale variations. (3) Insufficient multi-order contextual information capture: In real-world environments, objects do not exist in isolation. Their relationships—both semantic and spatial—are essential for accurate detection. Many current methods [9,10,11,12,13] fail to adequately capture multi-order contextual information, which includes both short-range and long-range dependencies between objects. This inability to model these relationships reduces the model’s robustness, leading to errors in object identification and spatial understanding. (4) Insufficient integration of channel information: Modern deep learning models rely on feature maps with multiple channels to represent different aspects of input data. However, many methods [14,15,16] struggle to aggregate this channel information effectively, leading to redundancy or loss of critical features. These models often fail to exploit inter-channel dependencies and overlook the rich semantic relationships between channels, which are crucial for accurately representing object features. (5) Singularity of depth estimation: Depth estimation from monocular images is inherently ambiguous, and relying on a single method for depth prediction often results in poor generalization across diverse and dynamic environments. Many modern monocular 3D object detection methods [17,18,19] depend on a single depth estimation strategy, making them unsuitable for complex, dynamic scenes. These methods typically lack sufficient robustness and generalization capabilities, failing to effectively address the challenges of depth estimation in varied scenarios. (6) Limitations of traditional attention mechanisms: Traditional attention mechanisms have shown success in enhancing feature representation by focusing on key regions of interest. However, existing methods [20,21] focus primarily on processing individual dimensions and fail to fully exploit the complementarity between features, limiting the representational power of feature semantics. Although the CBAM [22] model enhances feature expression by introducing a spatial attention mechanism, its fragmented computational paradigm struggles to effectively model the semantic dependencies between cross-dimensional features.

In addition to the aforementioned methods, similar issues still persist in recent works. YOLOBU [23] progressively analyzes local geometric and semantic information to improve detection accuracy. PDR [24] proposes the progressive depth regularization strategy to optimize depth estimation quality. MonoEdge [25] utilizes edge features from local perspectives to enhance detection performance in edge-blurred regions. MonoRCNN++ [26] introduces a multivariate probabilistic framework to better model the diversity of objects. MonoNeRD [27] enhances detection performance through implicit geometric modeling and volumetric rendering. Keypoint3D [28] projects the geometric center of 3D objects onto the 2D image plane as keypoints and integrates a self-adaptive elliptical Gaussian filter. DL-VFFA [29] captures the relationship between local details and the global context by improving the voxel processing process. AEPF [30] integrates an attention mechanism into the feature fusion strategy to enhance detection accuracy. These methods overlook the critical role of cross-dimensional feature interaction and multi-order contextual information modeling, particularly when lacking additional depth information. This limits the model’s performance in handling complex scenes, making it difficult to fully exploit the potential relationships between features.

In summary, we point out that existing methods lack cross-dimensional feature interaction and multi-order contextual information modeling, which limits the accuracy of detection. Therefore, we propose considering cross-dimensional feature interaction and multi-order contextual information modeling in detection to alleviate this issue. To tackle the aforementioned problems, we propose an adaptive multi-order perceptual aggregation framework, called MonoAMP. Evaluation of the KITTI [31] benchmark demonstrates that our method achieves significant performance improvements. Furthermore, to assess the generalization ability and robustness of the model, we conduct additional experiments on other datasets. The results show that our model consistently performs well across diverse datasets. We summarize the main contributions of our paper as follows:

1. We introduce the triplet attention mechanism and integrate it into the backbone network to alleviate the issue of insufficient cross-dimensional feature interaction.
2. We design an adaptive multi-order perception aggregation module, which dynamically models multi-order contextual information and effectively integrates channel information, addressing the issues of insufficient contextual information capture and inadequate channel information integration.
3. To further improve the accuracy of depth estimation in the absence of additional data, we propose an uncertainty-guided depth ensemble strategy.

## 2. Related Work

### 2.1. Anchor-Based Monocular 3D Detector

Accurately predicting the 3D poses of targets remains a primary challenge in monocular 3D detection due to the lack of depth information. Inspired by “anchor-based” 2D object detection methods, researchers have focused on regressing vehicle 3D pose parameters directly from images using convolutional neural networks. M3D-RPN [10] is a pioneering anchor-based framework that directly predicts the 3D position and dimensions of targets using 2D/3D geometric constraints and a 3D region generation network. MonoPair [32] innovatively introduces foreground–foreground and foreground–background anchor pairing modeling, enhancing the understanding of 3D spatial relationships through a parallelized pairing prediction network. Kinematic3D [33] further extends these works by reconstructing 3D scene dynamics from monocular video and improving 3D target localization in videos through stable object orientation decomposition and self-balanced 3D confidence estimation. MonoRCNN [8] generates dense anchors based on an RPN network, achieving end-to-end 3D localization through depth estimation and regional feature extraction. To address the feature misalignment issues in anchor-based methods, M3DSSD [34] introduces a two-stage feature alignment strategy, comprising target shape alignment and 2D/3D center alignment. However, these methods still rely on predefined anchors, which increase computational complexity and model limitations.

### 2.2. Center-Based Monocular 3D Detector

To reduce excessive reliance on anchors, researchers in 2019 proposed an “anchor-free” object detection framework, CenterNet [35]. This framework abstracts target regions as the centers of 2D bounding boxes and uses them as keypoints. Inspired by this, relevant researchers further extended it to monocular 3D detection. RTM3d [36] is a multi-scale feature pyramid 3D detection network that describes the 3D bounding box of a vehicle in image space using nine keypoints: eight vertices of the 3D box and one center point. It then utilizes geometric relationships across three-dimensional and two-dimensional views to determine the target’s spatial attributes. SMOKE [18] adopts a simpler architecture consisting of only two branches: keypoint estimation and 3D bounding box regression. Using a multi-step separation approach to construct 3D bounding boxes significantly improves network convergence and detection accuracy. MonoFlex [15] restores the target’s scale, position, and orientation by detecting the center point and incorporating geometric constraints. MonoDDE [37] further unveils cues from the object’s perspective based on this foundation. MonoDLE [11] highlights that although 2D bounding box prediction can be excluded, they remain crucial for forecasting 3D attributes. Research indicates that errors in depth estimation are the leading constraint on the accuracy of this detection task. Moreover, MonoCon [38] has demonstrated that incorporating additional learning information around the object can significantly enhance the model’s ability to generalize.

### 2.3. Transformer-Based Monocular 3D Detector

Recently, Transformer-based monocular 3D detection methods have made significant progress in enhancing global perception capabilities. For example, MonoDTR [39] incorporates depth position encoding to inject global depth information into the Transformer framework, which helps guide detection tasks and relies on LiDAR for supplementary supervision. In contrast, MonoDETR [40] leverages foreground target annotations to generate depth maps, thus providing depth guidance for the detection process. To improve computational effectiveness, MonoATT [41] employs an adaptive token transformer, which allocates detailed tokens to key areas. Despite these advances, Transformer-based monocular 3D detectors still suffer from challenges related to high computational complexity and slow inference times. These issues become particularly pronounced in real-world autonomous driving scenarios, where both the ability to synthesize global information and low-latency responses are crucial. At present, no solution exists that can effectively balance these two requirements.

## 3. Approach

### 3.1. Overall Framework of MonoAMP Network

**Figure 1:**
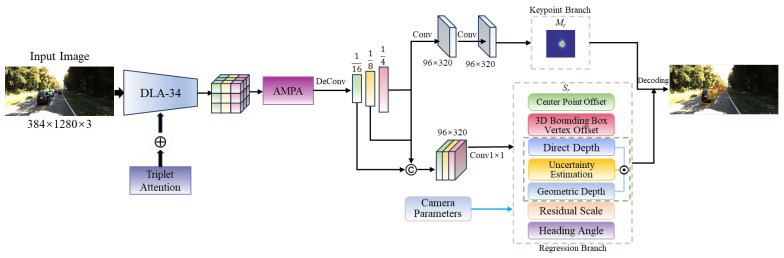
Overall framework of MonoAMP. The pink dashed box denotes the regression branch, the green denotes the uncertainty-guided depth ensemble, and the blue represents the keypoint branch. The symbol ⊕ represents the fusion operation, and © denotes the feature map concatenation operation.

The overall framework of our proposed method is illustrated in Figure 1. The multi-task detection head comprises two main branches. The keypoint branch estimates the likelihood of vehicle existence at the current location, generating a heatmap of vehicle center points. The regression branch predicts key geometric attributes, including depth, dimensions, and orientation of objects.

The backbone network utilizes DLA-34 [42] to extract features from images and incorporates triplet attention [43] to enhance the key feature extraction capability through collaborative interactions across different dimensions. The three-branch attention structure establishes cross-dimensional attention dependency modeling through rotational transformations and residual mappings. This structure constructs attention maps from three orthogonal planes and enhances the discriminative representation of features through adaptive attention weight computations based on dimensional decomposition, ultimately achieving efficient cross-dimensional attention interaction and fusion. The tail of the backbone network is connected to an adaptive multi-order perceptual aggregation (AMPA) module, which effectively aggregates multi-order contextual information, fully utilizes inter-channel correlations, and enhances the model’s feature selection capability.

Subsequently, a multi-scale feature hierarchy is constructed by hierarchically upsampling the features processed by the AMPA. We obtain feature maps with resolutions of 1/16, 1/8, and 1/4 of the input image size, respectively. We employ feature alignment operations to achieve a unified representation of multi-scale features, and feature concatenation is used to establish a joint semantic expression across multiple scales. During the feature fusion stage, we improve detection capability for multi-scale vehicles and address issues such as blurred object boundaries. For the heatmaps output by the keypoint branch, a set of response values is generated by applying a two-dimensional Gaussian kernel centered at the actual target point locations. The ground truth value of the vehicle center point on the heatmap corresponds to the pixel-level maximum response generated at the Gaussian kernel location. For depth prediction in the regression branch, an uncertainty-guided depth ensemble is designed, which adaptively fuses multiple depth estimation methods through weighted aggregation, thereby improving the model’s depth estimation precision and robustness. The regression vector of this network primarily includes center point offsets $R_{\mathrm{co}}$, 3D box vertex offsets $R_{3\mathrm{Dc}}$, vehicle residual scales $R_{\mathrm{res}}$, orientation angles $R_{\mathrm{ori}}$, direct depth $R_{\mathrm{dd}}$, uncertainty estimates $R_{\mathrm{unc}}$, and geometric depth $R_{\mathrm{kd}}$, among others.

### 3.2. Triplet Attention Mechanism

Existing attention mechanisms typically model feature relationships within a single dimension. SENet [44] adjusts the weights of different channels through channel attention, enhancing relevant features while suppressing irrelevant or redundant ones. However, SENet overlooks spatial information and fails to account for the correlations between different spatial locations. In contrast, CBAM [22] successfully demonstrates the importance of capturing both channel and spatial attention. Although CBAM improves performance, it does not consider cross-dimensional interactions. In monocular 3D object detection, the model needs to infer the spatial location, keypoints, pose, and size of objects from 2D images. Therefore, it is essential for the model to accurately capture both local and global features during the feature extraction phase. Unlike traditional 2D detection, it requires more refined feature representations, particularly for estimating the depth, spatial pose, and scale variations in objects. Therefore, the key to enhancing performance lies in effectively capturing the interaction between spatial and channel information. The triplet attention mechanism addresses cross-dimensional interactions through three parallel branches. Each branch focuses on modeling the interaction between the channel dimension (C) and the spatial dimension (H or W). This design enables complementary enhancement of information across dimensions. Theoretically, the triplet attention mechanism can significantly improve model performance, especially for monocular 3D object detection tasks that require fine-grained feature representation.

**Figure 2:**
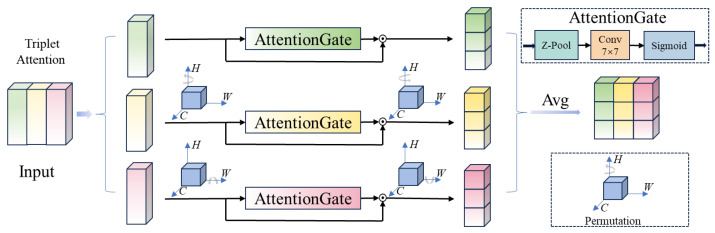
Illustration of TA.

In this paper, we introduce triplet attention [43] and integrate it into the backbone network to capture the mutual dependencies between the channel and spatial dimensions. Specifically, we integrate triplet attention (TA) into the deeper levels, Level 4 and Level 5, of the DLA-34 network for cross-dimensional information modeling. These layers typically extract high-level semantic features. It effectively enhances the fine-grained interactions between features, enabling the fine-grained feature representations required for the task. The TA strengthens cross-dimensional dependencies through parallel computation, combining rotational operations and residual transformations, as shown in Figure 2. In each branch, features of specific dimensions are compressed and extracted through Z-pool and convolution operations, while a Sigmoid function computes attention weights that determine the importance of features in each dimension. This mechanism enables the backbone network to enhance its focus on multi-dimensional features through a global self-attention perspective. It preserves the original information of the input features while enhancing cross-dimensional cooperation through the attention mechanism. All of this is achieved without significantly increasing computational overhead, thereby achieving a notable performance improvement.

### 3.3. The AMPA

**Figure 3:**
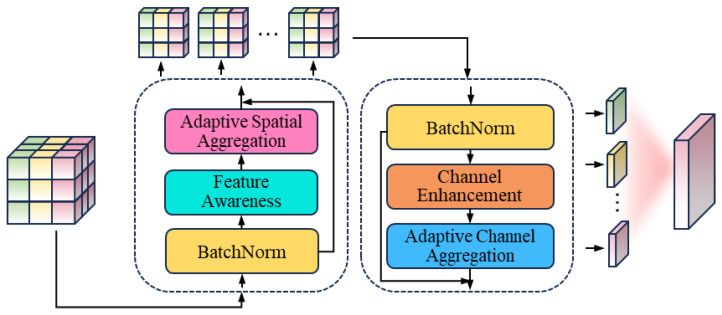
Illustration of AMPA.

The proposed AMPA is an architecture designed for aggregating multi-order contextual and channel information. The structure is shown in Figure 3. It aims to capture semantic correlations in contextual information and channel dependencies between features. The AMPA consists of four core sub-modules: feature perception module, adaptive spatial aggregation module, channel enhancement module, and adaptive channel aggregation module. The subsequent sections will delve into the specifics of each component’s construction and capabilities.

#### 3.3.1. Feature Awareness Module

**Figure 4:**
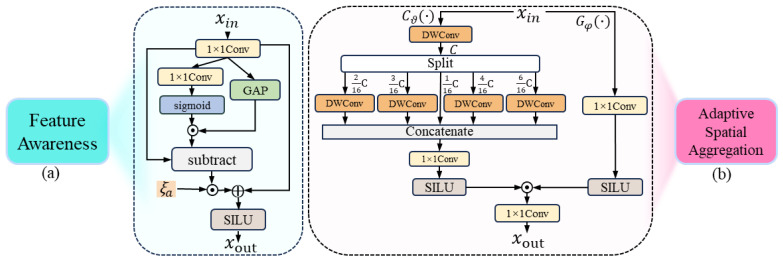
(**a**) The FA module. (**b**) The ASA module. The detailed structures corresponding to them are enclosed within their extended dashed boxes.

To balance the perception of local details and global semantic information, we design the feature awareness (FA) module. Figure 4a provides a comprehensive view of the structure. Specifically, it computes the difference between fine-grained features from the convolutional branch and dynamically weighted global features, highlighting the distinctions between detailed and global information. The complete procedure is outlined below:

(1) $$X_{\mathrm{out}}=\mathrm{SILU}\left(Y+\xi_{a}\cdot \left(Y-\sigma (f(Y))\cdot \mathrm{GAP}(Y)\right)\right)$$

where $Y=\mathrm{Conv}1\;\times \;1(X_{\mathrm{in}})$ represents the local features *Y* extracted from $X_{\mathrm{in}}$ using Conv1×1(·). GAP(Y) denotes the global features. The weight generation component for dynamic weighting of the global features is represented as σ(f(Y)), where *f* denotes Conv1×1(·). Furthermore, $\xi_{\alpha }$ is a learnable scaling factor, initialized to zero, for adjusting the weights of the difference term features.

#### 3.3.2. Adaptive Spatial Aggregation Module

Figure 4b shows the structure of the adaptive spatial aggregation (ASA). It is composed of two key functional pathways: $C_{\vartheta }\left(\cdot \right)$ context pathway and $G_{\varphi }\left(\cdot \right)$ gating pathway.

The $C_{\vartheta }\left(\cdot \right)$ context pathway is responsible for extracting multi-order contextual information. It employs depth-wise convolutions using varying dilation factors to comprehensively extract multi-order features. While controlling computational complexity, it expands the receptive field to capture multi-order contextual information from local to global. The $G_{\varphi }\left(\cdot \right)$ gating pathway adopts an adaptive gating mechanism. To adaptively aggregate features extracted from $C_{\vartheta }\left(\cdot \right)$, we use the adaptive activation function SILU [45]. It is specifically defined as: SILU(Y)=Y·σ(Y), where σ(Y) represents the sigmoid function. The SiLU possesses smooth gradients, which contribute to more stable gradient updates during the training process. Its derivative is:

(2) $$\frac{d}{dY}\mathrm{SILU}\left(Y\right)=\sigma \left(Y\right)+Y\cdot \sigma \left(Y\right)\cdot \left(1-\sigma \left(Y\right)\right)$$

Input features are processed through SILU to generate discriminative gating signals. Nonlinear transformations enable the capture of nonlinear dependencies between input features, facilitating the generation of more discriminative gating signals. SILU automatically adjusts the magnitude of the input features by multiplying *Y* with σ(Y). For positive and negative values, SILU exhibits distinct activation behaviors. Consequently, the gating signals can flexibly adapt to the specific characteristics of the input features, thereby enabling adaptive functionality. The importance of features at different orders is adaptively adjusted through dynamic gating signals.

The outputs from the gating pathway and the context pathway are fused to generate the final output. This enables the adaptive aggregation of multi-order context features. The entire process is instantiated as follows:

(3) $$X_{\mathrm{out}}=\underset{C_{\vartheta }\left(\cdot \right)}{\underset{︸}{f(\mathrm{SILU}\left(f\left(\mathrm{Concat}\left(X_{1},X_{2},X_{3},X_{4},X_{5}\right)\right)\right))}}⊙\underset{G_{\varphi }\left(\cdot \right)}{\underset{︸}{\mathrm{SILU}(Y)}}$$

where *f* denotes ${\mathrm{Conv}}_{1\times 1}\left(\cdot \right)$. $Y={\mathrm{Conv}}_{1\times 1}\left(X_{\mathrm{in}}\right)$ represents the input features of the gating pathway $G_{\varphi }\left(\cdot \right)$. $X_{i\in \{1,2,3,4,5\}}={\mathrm{DWConv}}_{\alpha_{i}C}\left(X_{\mathrm{in}}\right)$ represents the division of the input features $X_{\mathrm{in}}$ into multiple channel subsets with different ratios $\alpha_{i}$ for feature extraction, and $\sum_{i=1}^{5}\alpha_{i}C=C$.

#### 3.3.3. Channel Enhancement Module

**Figure 5:**
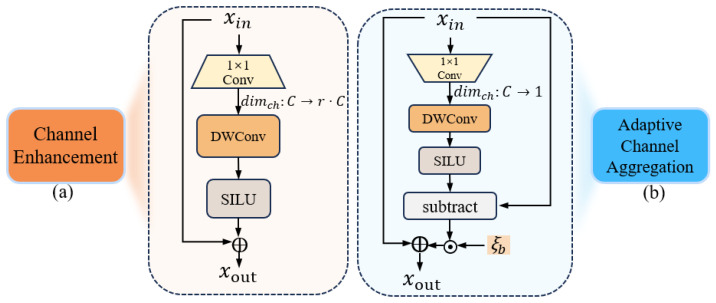
(**a**) The CE module. (**b**) The ACA module. The detailed structures corresponding to them are enclosed within their extended dashed boxes. The symbol → denotes projection operations that reduce or expand channel dimensions.

The channel enhancement (CE) module enhances the representational capability of input features and generates high-dimensional channel features. The CE module is shown in Figure 5a. First, it expands the number of feature map channels from the original dimension *C* to a higher dimension r·C. The SILU [45] activation function enhances discriminative feature representation through non-linear mapping. Specifically, the residual connection design enables the learning of residual mappings based on original features. The output is obtained through residual connection, which combines the input and transformed feature representations:

(4) $$X_{\mathrm{out}}=X_{\mathrm{in}}+\mathrm{SILU}\left(DWConv\left(UP\left(X_{\mathrm{in}}\right)\right)\right)$$

where $UP\left(X_{\mathrm{in}}\right)$ is implemented by a channel-expanding projection using ${\mathrm{Conv}}_{1\times 1}\left(\cdot \right)$: $R^{HW\times C}\to R^{HW\times r\cdot C}$.

#### 3.3.4. Adaptive Channel Aggregation Module

The adaptive channel aggregation (ACA) module addresses the key issues of insufficient channel dependency modeling and information redundancy in traditional methods. As shown in Figure 5b, it is designed with a channel compression and difference enhancement mechanism. First, a dimensionality reduction mapping is performed on the features along the channel dimension, capturing global dependencies between channels. Specifically, depth-wise convolutions are introduced to refine the feature representation of channels. The weighted difference information is fused with the original input features to generate richer output features:

(5) $$X_{\mathrm{out}}=X_{\mathrm{in}}+\xi_{b}\cdot \left(X_{\mathrm{in}}-\mathrm{SILU}\left(DWConv\left(UP\left(X_{\mathrm{in}}\right)\right)\right)\right)$$

where $Down\left(X_{in}\right)$ is implemented by a channel-reducing projection using ${\mathrm{Conv}}_{1\times 1}\left(\cdot \right)$: $R^{HW\times C}\to R^{HW\times 1}$. $\xi_{b}$ is a channel-level learnable scaling factor initialized to zero, used to adjust the weight of the difference term.

### 3.4. Uncertainty-Guided Depth Ensemble Strategy

For monocular 3D object detection, a single-depth prediction method often struggles to handle the complexities of various environments and scenarios. To enhance the robustness and accuracy of depth estimation, we propose an uncertainty-guided depth ensemble (UGDE). The depth estimation methods in this paper include direct regression of depth predictions and multiple geometric depth estimates derived from keypoints.

**Figure 6:**
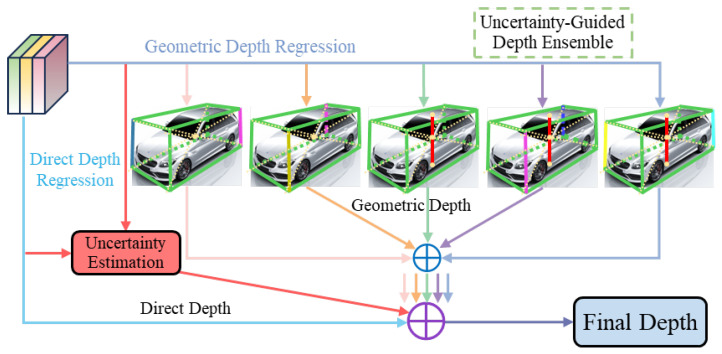
Illustration of the UGDE. In the geometric depth regression branch, we design five sets of geometric depths, including the vertical height of the vehicle center point and the heights of various diagonal combinations, and input them into the depth ensemble module.

As shown in Figure 6, our depth ensemble strategy combines multiple depth estimation methods and adaptively weights different predictions based on uncertainty, generating more reliable depth estimates. Each depth prediction $Z_{i}$ has a corresponding weight $w_{i}$, which represents the model’s confidence in that prediction. Through uncertainty-based weighted averaging, predictions with higher confidence are given greater weight, dominating the depth ensemble. The computation formula for the ensemble is as follows:

(6) $$Z_{\mathrm{ens}}=\frac{\sum_{i=1}^{N}Z_{i}\cdot w_{i}}{\sum_{i=1}^{N}w_{i}}$$

where the weight term $w_{i}$ is defined as $\frac{1}{\varphi_{i}}$. Estimates with smaller uncertainty $\varphi_{i}$ are given greater weights, while those with larger uncertainty are given lower weights.

**Figure 7:**
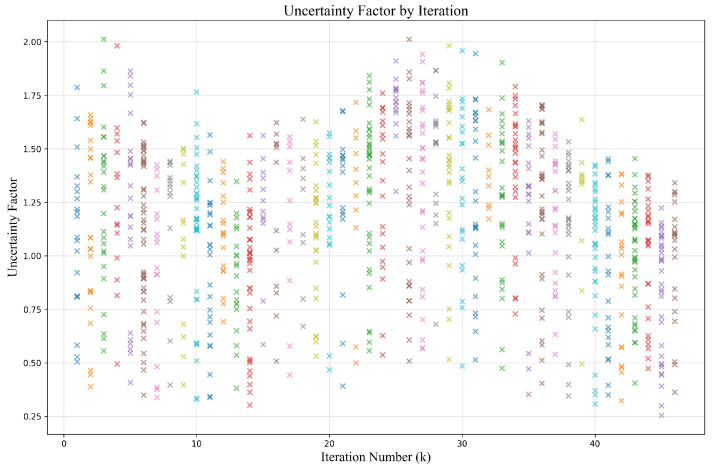
The uncertainty factor visualization. We present the values of the uncertainty factor at different iterations during the training process. We use different colors to more intuitively distinguish the uncertainty factor at different iterations.

The uncertainty factor is computed from the model’s regression feature maps. Specifically, it is estimated based on the input features and the model’s performance on that input. During the forward pass, the regression feature maps contain the depth predictions along with other related features. We extract the features corresponding to the target locations (i.e., points of interest, POI) from these maps and obtain the specific regression results for each location using the corresponding indices. The uncertainty factor, which plays a critical role in depth estimation, is embedded in a dedicated channel of the regression feature map. This value indicates the reliability of the model’s depth predictions in certain regions. In practice, the uncertainty factor is constrained within a predefined range to prevent it from exceeding a reasonable limit, ensuring that the model’s uncertainty predictions remain both precise and adjustable. The visualization of the uncertainty factor during the training process is shown in Figure 7. The uncertainty factor is influenced by various factors, such as differences in input images or scene conditions, which can lead to variations in the uncertainty. When the training data include complex, occluded, or blurred scenes, the model may exhibit higher uncertainty in such situations. Furthermore, the model’s architecture and training process also play a crucial role in determining the magnitude of the uncertainty factor.

### 3.5. Multi-Task Loss Function Design

Based on the designed network output, the loss function consists of seven components: center point classification loss $L_{c}$, 3D bounding box vertex offset loss $L_{3Dc}$, center point offset loss $L_{co}$, direct regression depth loss $L_{dd}$, geometric depth loss $L_{kd}$, vehicle 3D residual scale loss $L_{res}$, and heading angle loss $L_{ori}$. The center point classification loss $L_{c}$ is computed using focal loss [46]:

(7) $$L_{c}=-\frac{1}{N}\sum_{i=1}^{h}\sum_{j=1}^{w}\left\{\begin{array}{cc} \;{\left(1-{\hat{V}}_{c_{ij}}\right)}^{\alpha }\log\left({\hat{V}}_{c_{ij}}\right), & V_{c_{ij}}=1\\ {\left(1-V_{c_{ij}}\right)}^{\beta }{\hat{V}}_{c_{ij}}^{\alpha }\log\left(1-{\hat{V}}_{c_{ij}}\right), & \mathrm{otherwise} \end{array}\right.$$

where α and β are hyperparameters that adjust the weights of the positive and negative sample losses. *N* indicates the quantity of positive samples. ${\hat{V}}_{c_{ij}}$ represents the predicted response value at (*i*, *j*). $V_{c_{ij}}$ represents the true response value at (*i*, *j*) for the Gaussian kernel function $e^{\frac{x^{2}+y^{2}}{2\sigma^{2}}}$, and σ is the standard deviation of the Gaussian distribution.

The center point offset loss $L_{co}$ and 3D bounding box vertex offset loss $L_{3Dc}$ are trained using L1 Loss:

(8) $$L_{co},L_{3Dc}=-\frac{1}{N}\sum_{i=1}^{h}\sum_{j=1}^{w}\left|\delta_{p}-\left(\frac{P}{R}-\tilde{P}\right)\right|$$

where *P* represents the actual target point coordinates. *R* is the downsampling factor. $\delta_{p}$ denotes the true coordinate offset. $\tilde{P}=\left\lfloor \frac{P}{R}\right\rfloor (\left\lfloor \cdot \right\rfloor$ denotes the floor operation). $\frac{p}{R}-\tilde{P}$ represents the predicted coordinate offset.

For the vehicle 3D residual scale $L_{res}$, each regression quantity is evaluated using the Smooth L1 Loss [47]:

(9) $$L_{\mathrm{res}}=\sum_{\delta \in (l,w,h)}\mathrm{Smooth}\;L1\left(\Delta \delta \right)$$

where *h*, *w*, and *l* represent the real height, width, and length dimensions of the vehicle, respectively. Δδ represents the numerical difference between the ground truth and predicted values.

The heading angle regression loss $L_{ori}$ is predicted by applying the Multi-Bin [48] method. In our approach, we define four bins with centers at $\left[0,\frac{\pi }{2},\pi ,-\frac{\pi }{2}\right]$. The total loss for Multi-Bin orientation estimation is:

(10) $$L_{ori}=L_{conf}+w\times L_{loc}$$

The confidence loss $L_{conf}$ is defined as the softmax loss for the confidence of each interval. The calculation formula for the localization loss $L_{loc}$ is as follows:

(11) $$L_{loc}=-\frac{1}{m_{\theta^{*}}}\sum \cos\left(\theta^{*}-b_{i}-\Delta \theta_{i}\right)$$

where $m_{\theta^{*}}$ denotes the number of intervals covering the true heading angle $\theta^{*}$. $b_{i}$ represents the center angle of interval *i*. $\Delta \theta_{i}$ denotes the correction required for the center of interval *i*.

The direct regression depth loss $L_{dd}$ is designed to quickly capture depth features by directly regressing the object depth information. The network output $Z_{o}$ is first transformed into absolute depth using the inverse sigmoid function to reduce the influence of the output range on predictions.

(12) $$Z_{r}=\frac{1}{\sigma \left(Z_{o}\right)+\varepsilon }-1$$

where $\sigma \left(x\right)$ is defined as $\sigma \left(x\right)=\frac{1}{1+e^{-x}}$. The term ε represents a small constant that is introduced to ensure the stability of the direct depth calculation process. Meanwhile, by incorporating uncertainty modeling, the model can adaptively adjust the loss value when it has low confidence in depth predictions.

(13) $$L_{\mathrm{dep}}=\frac{\left|Z_{r}-Z^{*}\right|}{\varphi_{\mathrm{dd}}}+\log\left(\varphi_{\mathrm{dd}}\right)$$

where $Z_{r}$ denotes the predicted depth. $Z^{*}$ denotes the ground truth depth. $\log(\varphi_{\mathrm{dd}})$ is used to prevent the model from increasing uncertainty to avoid loss. $\varphi_{dd}$ is the predicted depth uncertainty.

The keypoint-based geometric depth loss $L_{kd}$ estimates depth by leveraging the geometric relationships of the object’s keypoints. It computes the center depth $Z_{c}$ and the depths of four 3D bounding box diagonals, $Z_{d_{1}}$, $Z_{d_{2}}$, $Z_{d_{3}}$, and $Z_{d_{4}}$. The loss function for geometric depth estimation also uses L1 Loss and incorporates uncertainty modeling:

(14) $$L_{kd}=\sum_{k\in \{c,d_{1},d_{2},d_{3},d_{4}\}}\left[\frac{|Z_{k}-Z^{*}|}{\varphi_{k}}+Y_{in}\left(Z_{k}\right)\log\left(\varphi_{k}\right)\right]$$

where $Z_{k}$ represents the depth calculated based on the geometric relationships of the keypoints. $\varphi_{k}$ denotes the uncertainty of the depth. $Y_{in}\left(Z_{k}\right)$ is an indicator function that determines whether the keypoints used for depth calculation are visible. When the keypoints are not visible, the model allows the impact of the loss to be reduced by assigning a larger uncertainty.

Based on the above, the comprehensive loss function of the network, Loss, is formulated in the manner presented below:

(15) $$Loss=r_{1}L_{c}+r_{2}L_{co}+r_{3}L_{3Dc}+r_{4}L_{dd}+r_{5}L_{kd}+r_{6}L_{res}+r_{7}L_{ori}$$

where $r_{1}$, $r_{2}$, $r_{3}$, $r_{4}$, $r_{5}$, $r_{6}$, and $r_{7}$ are the balance factors between the individual loss functions, with $r_{1}+r_{2}+r_{3}+r_{4}+r_{5}+r_{6}+r_{7}=1$.

## 4. Experiments

### 4.1. Dataset

**KITTI.** This study primarily employs the KITTI 3D Object dataset [31], a widely adopted benchmark in autonomous driving research, particularly for 3D object detection tasks. It provides 7481 training images and 7518 testing images, all captured from real-world driving scenarios and accompanied by detailed 3D annotations. Since the test image annotations are not publicly available, we follow the strategy described in [49], dividing the 7481 training images into 3712 for training and 3769 for validation. This split ensures the independence of the training and validation sets, thereby enhancing the reliability of the experimental outcomes. Based on the degree of truncation, bounding box height, and occlusion, the levels of difficulty are defined as easy, moderate, and hard. Table 1 presents the specific classification criteria.

**Waymo.** Waymo [50] categorizes objects into Level 1 and Level 2 based on the count of LiDAR points contained within their 3D bounding boxes. Evaluations are performed across three distance ranges: [0,30), [30,50), and [50,∞) meters. Waymo employs the $APH_{3D}$ and $AP_{3D}$ metrics as standard evaluation measures. $APH_{3D}$ is an extension of $AP_{3D}$ that integrates heading angle information.

**NuScenes.** NuScenes [51] includes training images (28,130) and validation images (6019), all recorded using the front-facing camera. To evaluate the model’s generalization capability, we employ a validation subset for cross-dataset evaluation.

### 4.2. Implementation Details

The experimental setup is summarized in Table 2. The proposed approach is developed on PyTorch 2.0.1. All input images were resized uniformly to 384 × 1280. The only data augmentation employed is random horizontal flipping of the images. The model is trained with the AdamW [52] optimizer, initialized at a learning rate of 0.0003. This process utilizes a batch size of 8. The training is conducted on an individual NVIDIA A800 GPU, performing a total of 46k iterations. Additionally, the learning rate is adjusted by reducing it to one-tenth of its value at 37k and 42k iterations.

### 4.3. Evaluation Metrics

This study aligns with current mainstream research, with a particular focus on the performance of models in vehicle category detection tasks. For performance evaluation, Average Precision (AP) is used as the primary metric. After that, the 40 recall positions (RPs) are considered to enhance the comprehensiveness of the evaluation, following MonoDIS [6]. The Intersection-over-Union (IoU) threshold is set to 0.7. It ensures the rigor and consistency of the evaluation process by accurately measuring the overlap between ground truth and predicted values. This multi-level evaluation strategy aims to comprehensively reflect the model’s performance across varying detection difficulties.

### 4.4. Quantitative Results

**Results of KITTI test set.** We provide a comprehensive analysis comparing our novel approach with leading methodologies developed in recent years, evaluated on the widely used KITTI test set. MonoGRNet [9], MonoDIS [6], M3D-RPN [10], SMOKE [18], MonoDLE [11], MonoRCNN [8], MonoFlex [15], and MonoGround [53] are currently popular standard monocular 3D detectors, which use only RGB images as the network’s sole input without introducing additional data (e.g., LiDAR or CAD). Additionally, DDMP-3D [7], UR3D [54], Kinematic3D [33], AutoShape [55], MonoRUn [56], CaDDN [57], and MonoDTR [39] incorporate auxiliary data to guide detector training.

The data in Table 3 demonstrate that our method achieves significant improvements. Notably, it shows superior accuracy in detecting objects at the intermediate level, which is a crucial aspect of monocular 3D object detection. Quantitative analysis demonstrates that our method achieves ${AP}_{3D}$ scores of 23.89%, 16.80%, and 14.06%, and ${AP}_{BEV}$ scores of 33.61%, 24.47%, and 21.08%. In contrast to existing methods, our novel approach demonstrates clear superiority, outperforming several methods that do not use auxiliary data, such as M3D-RPN [10], SMOKE [18], MonoDLE [11], MonoRCNN [8], MonoFlex [15], MonoGround [53], and MonoJSG [4]. It also surpasses some methods that utilize auxiliary data, including DDMP-3D [7], Kinematic3D [33], AutoShape [55], CaDDN [57], and MonoDTR [39]. Additionally, relative to the advanced Transformer-based detector MonoDETR [40], our method outperforms it on most performance metrics while ensuring real-time functionality.

The improvements achieved by our method can be attributed to its enhanced ability to learn contextual relationships and spatial dependencies between objects. By focusing on more precise depth estimation and refining the spatial interaction between features, our model better captures fine-grained information about object positioning and structure. This enhances the accuracy of scene understanding, leading to significant improvements in localization and depth precision. The model’s effective fusion of multi-order contextual information allows it to better represent complex scene dynamics, resulting in stable performance gains across both ${AP}_{3D}$ and ${AP}_{BEV}$ metrics.

**Results of Waymo validation set.** We evaluate the generalization ability of our MonoAMP method on the Waymo dataset. Table 4 shows that MonoAMP surpasses state-of-the-art methods in most evaluation metrics. It delivers excellent performance across various thresholds, particularly for objects at moderate distances.

**Cross-dataset evaluation.** We conduct cross-dataset evaluation experiments to further assess the model’s generalization ability. We use our KITTI validation model to evaluate the mean absolute error (MAE) of depth on nuScenes [51] frontal validation and KITTI validation images. As shown in Table 5, our MonoAMP outperforms GUPNet [18]. Meanwhile, our results are very close to DEVIANT [60], whose method is equivalent to depth transformation and demonstrates robustness to variations in data patterns.

**Efficiency.** In evaluating the efficiency of the proposed model, we emphasize computational cost, which is critical for real-time applications. We provide a clear comparison with existing state-of-the-art methods by reporting the Giga Floating-Point Operations per Second (GFLOPs), Runtime (ms), and $AP_{3D}$ for our model. As shown in Table 6, we present performance data for these methods, including the widely used transformer-based approaches, MonoDTR [40] and MonoDETR [41]. MonoAMP achieves competitive detection performance while maintaining lower computational cost.

### 4.5. Ablation Study

This section presents ablation studies to assess the contribution of each module in the proposed method and its impact on overall performance. MonoDLE [11] is the baseline method of the ablation study. Specifically, we conducted experiments by incrementally adding key modules to the baseline method and analyzing their impact on overall performance with a rigorous evaluation based on experimental metrics. All experiments were conducted under identical conditions to ensure the comparability of results. The results of the ablation study are presented in Table 7.

The ablation study shows that each proposed module greatly enhances the performance of the baseline model in monocular 3D object detection. After incorporating the TA into the baseline model, the model’s ability to allocate attention to critical regions is significantly improved. The FA module enhances the model’s perception of key spatial features through an adaptive feature enhancement mechanism. The ASA module captures multi-level contextual information, improving the model’s adaptability to complex environments. The CE module optimizes the inter-channel dependency, enhancing the model’s performance with multi-channel features. By adding the ACA module, the aggregation of channel features is optimized, enabling the model to capture important channel information more accurately, thereby improving overall performance. The UGDE module adaptively fuses multiple depth prediction strategies, enabling the model to exhibit strong robustness and accuracy in monocular 3D object detection.

Quantitative analysis shows that, for the easy, moderate, and hard scenarios of the monocular 3D object detection task, the proposed method improves the ${AP}_{3D}$ by 5.13, 3.78, and 3.06 percentage points, respectively, and the ${AP}_{BEV}$ by 5.94, 4.42, and 4.14 percentage points compared to the baseline method. These experimental results fully validate the effectiveness and synergistic effects of each module and demonstrate the significant advantages of the proposed method in enhancing detection performance and improving model robustness.

**Efficiency of each component.** To further evaluate the efficiency and performance of the key components in this study, we conduct experiments to analyze the contribution of each component to the overall performance. Table 8 illustrates that each component achieves performance improvements while maintaining relatively low computational resource consumption.

### 4.6. Visualization Analysis

**Figure 8:**
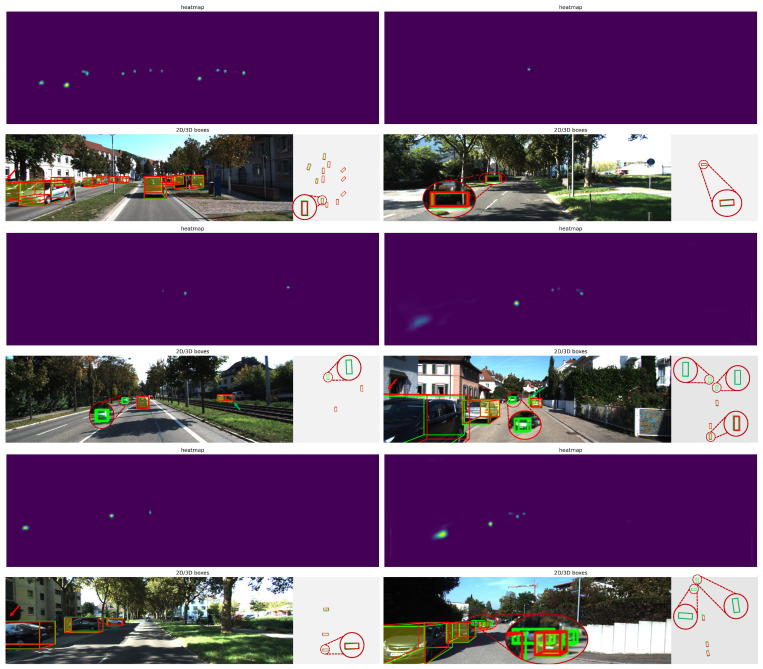
Visualization results of the MonoAMP experiment. We provide the detection results under various road conditions. The red arrows primarily highlight instances of vehicle truncation.

In this section, we visualize the detection results in various scenarios to comprehensively assess the performance of the proposed approach, including heatmaps, 3D bounding boxes, and bird’s-eye views. As shown in Figure 8, we compare the predicted results of the proposed approach with actual scenes, demonstrating its performance in complex scenarios such as dense vehicle crowds, varying object sizes, occlusions, and truncated targets. The red denotes the ground truth, while the green indicates the predicted bounding boxes.

The heatmap shows that the model generates clear responses in densely populated vehicle areas. Our method still provides reliable responses even in distant scenarios or when occlusions or truncations occur. For vehicles that are close and unobstructed, the method generates 3D prediction bounding boxes that closely match the ground truth. In cases of severe vehicle truncation, such as the vehicle at the image edge marked by the red arrow, traditional methods often struggle to accurately estimate its depth or shape. In contrast, we effectively mitigate the depth ambiguity issue through the UGDE, successfully generating 3D bounding boxes that closely match the ground truth. The bird’s-eye view further demonstrates the method’s accurate localization and identification of severely truncated objects. Our method also detects small, distant vehicles that are not annotated in the dataset, as shown by the green 3D prediction bounding boxes highlighted by the magnifying glass in the figure, along with their corresponding bird’s-eye view. This shows that our model exhibits strong generalization and perception abilities when handling unannotated objects. Overall, the proposed method demonstrates significant accuracy and robustness in complex traffic scenarios, including dense object detection, small distant object recognition, and occlusion or truncated object detection.

## 5. Conclusions

In this paper, we reveal the limitations of existing monocular 3D object detection methods, particularly in terms of cross-dimensional feature attention mechanisms and multi-order contextual information modeling. We propose MonoAMP, a novel framework with the AMPA. By employing cross-dimensional attention interactions, we fully exploit both spatial and channel information of the features, enhancing the expression of key features. Additionally, we design AMPA that enhances the model’s ability to adaptively aggregate multi-order contextual information, effectively alleviating the issue of multi-scale objects. To achieve more stable and accurate depth predictions, we propose an uncertainty-guided adaptive depth ensemble strategy. This strategy dynamically allocates weights to different depth estimation methods, ensuring that high-confidence depth values contribute more significantly during the fusion process. Experiments on the KITTI benchmark show that MonoAMP performs exceptionally well in complex scenarios. Whether in high-density traffic scenarios or challenging environments with occlusions and truncation, it consistently demonstrates excellent detection performance. This validates its potential value and broad applicability in real-world applications. Although MonoAMP demonstrates robust performance, its accuracy remains a challenge in meeting the stringent demands of real-world autonomous driving applications. Future research will aim to enhance both detection precision and computational efficiency, ensuring greater applicability in complex and dynamic environments. By addressing these challenges, it holds significant potential to advance the field of monocular 3D object detection.

## Figures and Tables

**Table 1:** Difficulty level classification criteria of the KITTI Dataset.

Level	Min. Bounding Box Height	Max. Truncation	Max. Occlusion Level
Easy	40 Px	15%	Fully visible
Moderate	25 Px	30%	Partly occluded
Hard	25 Px	50%	Difficult to see

**Table 2:** Experimental configuration.

Item	Configuration or Version
CPU	Intel Xeon Gold 6336Y
Operating System	Ubuntu 20.04.3
GPU	NVIDIA A800
GPU Memory	80 GB
CUDA	11.8

**Table 3:** Comparative performance of different methods. $AP_{40}$ (%) metrics for the car category on the KITTI test set were evaluated using a 0.7 IoU threshold. Methods are categorized based on the extra data they utilize. Within each category, methods are sorted according to the $AP_{3D}$ results at the Moderate difficulty level (Mod.). Notably, the top-performing outcomes are presented in boldface, and the subsequent best are indicated with an underline.

Methods, Venues	Extra	${\mathrm{AP}}_{3D}$, Test (Car)				${\mathrm{AP}}_{\mathrm{BEV}}$, Test (Car)			Time
		Eazy	Mod.	Hard		Eazy	Mod.	Hard	(ms)
Kinematic3D [33], ECCV2020	Video	19.07	12.72	9.17		26.69	17.52	13.10	120
UR3D [54], ECCV2020	Depth	15.58	8.61	6.00		21.85	12.51	9.20	120
DDMP-3D [7], CVPR2021	Depth	19.71	12.78	9.80		28.08	17.89	13.44	180
AutoShape [55], ICCV2021	CAD	22.47	14.17	11.36		30.66	20.08	15.59	50
MonoRUn [56], CVPR2021	LiDAR	19.65	12.30	10.58		27.94	17.34	15.24	70
CaDDN [57], CVPR2021	LiDAR	19.17	13.41	11.46		27.94	18.91	17.19	630
MonoDTR [39], CVPR2022	LiDAR	21.99	15.39	12.73		28.59	20.38	17.14	37
MonoNeRD [27], ICCV2023	LiDAR	20.64	15.44	13.99		29.03	22.03	19.41	-
MonoGRNe [9], AAAI2019	None	9.61	5.74	4.25		18.19	11.17	8.73	60
MonoDIS [6], CVPR2019	None	10.37	7.94	6.40		17.23	13.19	11.12	100
M3D-RPN [10], ICCV2019	None	14.76	9.71	7.42		21.02	13.67	10.23	160
SMOKE [18], CVPRW2020	None	14.03	9.76	7.84		20.83	14.49	12.75	30
MonoDLE [11], CVPR2021	None	17.23	12.26	10.29		24.79	18.89	16.00	40
MonoRCNN [8], ICCV2021	None	18.36	12.65	10.03		25.48	18.11	14.10	70
MonoRCNN++ [26], WACV2023	None	20.08	13.72	11.34		-	-	-	-
MonoFlex [15], CVPR2021	None	19.94	13.89	12.07		28.23	19.75	16.89	35
MonoGround [53], CVPR2022	None	21.37	14.36	12.62		30.07	20.47	17.74	30
MonoEdge [25], WACV2023	None	21.08	14.47	12.73		28.80	20.35	17.75	-
MonoJSG [4], CVPR2022	None	24.69	16.14	13.64		32.59	21.26	18.18	42
PDR [24], TCSVT2023	None	23.69	16.14	$\underline{13.78}$		31.76	21.74	$\underline{18.79}$	-
YOLOBU [23], RA-L2023	None	22.43	16.21	13.73		30.54	21.66	18.64	-
MonoDETR [40], ICCV2023	None	**25.00**	16.47	13.58		$\underline{33.60}$	$\underline{22.11}$	18.60	43
MonoUNI [58], NeurIPS2024	None	$\underline{24.75}$	$\underline{16.73}$	13.49		24.51	17.18	14.01	-
MonoAMP(Ours)	None	23.89	**16.80**	**14.06**		**33.61**	**24.47**	**21.08**	35

**Table 4:** Evaluation results of Waymo validation set.

Difficulty	Method	Extra	${\mathrm{APH}}_{3D}/{\mathrm{AP}}_{3D}$, Val (Car, IoU = 0.7)			
			0–30 m	30–50 m	50 m–∞	All
Level_1	PatchNet [20]	Depth	1.63/1.67	0.12/0.13	0.03/0.03	0.39/0.39
	PCT [59]	Depth	3.15/3.18	0.27/0.27	0.07/0.07	0.88/0.89
	CaDDN [57]	LiDAR	14.43/15.54	1.45/1.47	0.10/0.10	4.99/5.03
	M3D-RPN [10]	None	1.10/1.12	0.18/0.18	0.02/0.02	0.34/0.35
	GUPNet [18]	None	6.11/6.15	0.80/0.81	0.03/0.03	2.27/2.28
	DEVIANT [60]	None	6.90/6.95	**0.98**/**0.99**	0.02/0.02	2.67/2.69
	MonoUNI [58]	None	8.50/8.61	0.86/0.87	0.12/0.13	3.16/3.20
	MonoAMP(Ours)	None	**8.83**/**8.95**	$\underline{0.94}$/$\underline{0.95}$	**0.14**/**0.15**	**3.32**/**3.35**
Level_2	PatchNet [20]	Depth	1.63/1.67	0.11/0.13	0.03/0.03	0.36/0.38
	PCT [59]	Depth	3.15/3.18	0.26/0.27	0.07/0.07	0.66/0.66
	CaDDN [57]	LiDAR	14.38/14.50	1.41/1.42	0.09/0.09	4.45/4.49
	M3D-RPN [10]	None	1.10/1.12	0.17/0.18	0.02/0.02	0.33/0.35
	GUPNet [18]	None	6.08/6.13	0.77/0.78	0.02/0.02	2.12/2.14
	DEVIANT [60]	None	6.87/6.93	**0.94**/**0.95**	0.02/0.02	2.50/2.52
	MonoUNI [58]	None	8.48/8.59	0.84/0.85	0.12/0.12	3.00/3.04
	MonoAMP(Ours)	None	**8.81**/**8.92**	$\underline{0.91}$/$\underline{0.92}$	**0.13**/**0.14**	**3.17**/**3.20**
**Difficulty**	**Method**	**Extra**	**${\mathrm{APH}}_{3D}/{\mathrm{AP}}_{3D}$, Val (Car, IoU = 0.5)**			
			**0–30 m**	**30–50 m**	**50 m–∞**	**All**
Level_1	PatchNet [20]	Depth	9.75/10.03	0.96/1.09	0.18/0.23	2.74/2.92
	PCT [59]	Depth	14.54/14.70	1.75/1.78	0.39/0.39	4.15/4.20
	CaDDN [57]	LiDAR	44.46/45.00	9.11/9.24	0.62/0.64	17.54/17.31
	M3D-RPN [10]	None	10.70/11.14	2.09/2.16	0.21/0.26	3.63/3.79
	GUPNet [18]	None	24.59/24.78	4.78/4.84	0.22/0.22	9.94/10.02
	DEVIANT [60]	None	26.64/26.85	**5.08**/**5.13**	0.18/0.18	10.89/10.98
	MonoUNI [58]	None	26.30/26.63	3.98/4.04	0.55/0.57	10.73/10.98
	MonoAMP(Ours)	None	**27.13**/**27.47**	$\underline{4.86}$/$\underline{4.92}$	**0.79**/**0.81**	**11.17**/**11.29**
Level_2	PatchNet [20]	Depth	9.73/10.01	0.97/1.07	0.16/0.22	2.28/2.42
	PCT [59]	Depth	14.51/14.67	1.71/1.74	0.35/0.36	4.15/4.03
	CaDDN [57]	LiDAR	44.33/44.87	8.86/8.99	0.55/0.58	16.28/16.51
	M3D-RPN [10]	None	10.67/11.12	2.04/2.12	0.20/0.24	3.46/3.61
	GUPNet [18]	None	24.50/24.69	4.62/4.67	0.19/0.19	9.31/9.39
	DEVIANT [60]	None	26.54/26.75	**4.90**/**4.95**	0.16/0.16	10.20/10.29
	MonoUNI [58]	None	26.24/26.57	3.89/3.95	0.51/0.53	10.24/10.38
	MonoAMP(Ours)	None	**27.06**/**27.40**	$\underline{4.78}$/$\underline{4.84}$	**0.76**/**0.78**	**10.65**/**10.78**

**Table 5:** Cross-dataset evaluation on nuScenes frontal validation and KITTI validation datasets.

Method	nuScenes Frontal Val (Depth MAE)					KITTI Val (Depth MAE)			
	All	0–20 m	20–40 m	40 m–∞		All	0–20 m	20–40 m	40 m–∞
M3D-RPN [10]	2.67	0.94	3.06	10.36		1.26	0.56	1.33	2.73
MonoRCNN [8]	2.39	0.94	2.84	8.65		1.14	0.46	1.27	2.59
GUPNet [18]	1.45	0.82	$\underline{1.70}$	6.20		0.89	0.45	1.10	1.85
DEVIANT [60]	**1.26**	$\underline{0.76}$	**1.60**	**1.26**		0.87	0.40	1.09	1.80
MonoAMP	$\underline{1.42}$	**0.68**	1.76	$\underline{4.95}$		**0.82**	**0.37**	**0.90**	**1.78**

**Table 6:** Efficiency comparison of different methods.

Method	MonoDTR	GUPNet	MonoDETR	MonoDLE	MonoAMP
**${\mathrm{AP}}_{3D}$ Mod.**	15.39	14.02	16.47	12.26	**16.80**
**Runtime ↓**	37	34	38	40	35
**GFlops ↓**	120.48	62.32	62.12	79.12	**61.78**

**Table 7:** Ablation study results. The best results are emphasized in bold.

Methods	${\mathrm{AP}}_{3D}$, Test (Car)				${\mathrm{AP}}_{\mathrm{BEV}}$, Test (Car)		
	Eazy	Mod.	Hard		Eazy	Mod.	Hard
Baseline	17.23	12.26	10.29		24.79	18.89	16.00
Baseline + TA	18.45	13.57	11.13		26.03	19.52	17.05
Baseline + TA + FA	18.93	14.05	11.56		27.24	20.36	17.83
Baseline + TA + FA + ASA	19.54	14.71	12.15		28.32	21.02	18.65
Baseline + TA + FA + ASA + CE	20.01	15.03	12.43		29.12	21.67	18.97
Baseline + TA + AMPA (FA + ASA + CE + ACA)	21.31	15.57	12.81		29.93	22.13	19.34
Baseline + TA + AMPA (FA + ASA + CE + ACA) + UGDE	**22.36**	**16.04**	**13.35**		**30.73**	**23.31**	**20.14**

**Table 8:** Efficiency of each component based on the baseline model.

Methods	Params (M)	GFLOPs	${\mathrm{AP}}_{3D}$, Test (Car)				${\mathrm{AP}}_{\mathrm{BEV}}$, Test (Car)		
			Easy	Mod.	Hard		Easy	Mod.	Hard
Baseline	20.32	79.12	17.23	12.26	10.29		24.79	18.89	16.00
Baseline + TA	20.32	79.15	18.45	13.57	11.13		26.03	19.52	17.05
Baseline + AMPA (FA + ASA + CE + ACA)	20.68	79.53	**19.36**	**14.35**	**12.03**		**27.96**	**21.13**	**18.35**
Baseline + UGDE	20.32	79.13	18.65	13.67	11.56		26.31	19.35	16.97

## Data Availability

The data underlying this study’s findings are included in the article. The KITTI dataset is available at http://www.cvlibs.net/datasets/kitti/(accessed on 25 January 2025).
